# Children’s Winter Dress

**Published:** 1871-01

**Authors:** 


### CHILDREN'S WINTER DRESS.

There is a fashionable way and a healthful way to dress a child. Mothers generally pay more attention to the former than to the latter. It is doubtless very pretty and becoming to dress a little girl in short skirts, covering her daintily-shaped ankle and handsome limb with a thin, silken, or cotton stocking, encasing her foot in a thin-soled and exquisitely shaped shoe, while her shoulders are loaded with cloak, furs and scarf. She looks well—presents an elegant appearance, in fact, and the mother is pleased thereat. But if that same mother should be attacked by some idiosyncratic tailor, by the wayside, and through his manipulations should suffer the curtailment of her petticoats, all around about, (as is graphically related in some juvenile work on æsthetics, the title of which was well known to us in our youthful days, but has now, alas, escaped us),—until her poor, shivering knees were exposed to the gentle zephyrs of a bright December day, it strikes us that if she did not freeze to death beforeshe reached home, she would have some slight appreciation of the condition of her child, and dress her thereafter, in a less fashionable, but more comfortable style.

It is really distressing to witness this manner of dressing children during the winter months. No grown person could be comfortable for a moment in such a rig, and it is only from constant exercise in running, that children so clad, can secure any degree of comfort, while upon the street. Dressing their extremities so thinly is not only uncomfortable but unhealthful, as well. When they run, becoming heated in play, and then sit or stand in the open air, the blood is driven rapidly from the extremities to the trunk, endangering the little ones to congestion of the lungs and mucus surfaces, when they are said to have a "bad cold."

See that your children wear snugly-fitting, woolen,or canton-flannel drawers next their skin; over this the stocking may be drawn, and in the colder days, woolen leggings should be worn over all. Let their shoes be thick and covered by warm overshoes; their limbs may not look so neatly, but they will certainly be comfortable, and the corresponding improvement in the healths of your children will more than repay you for your temporary mortification at their unfashionable appearance.

### TRUSSES.

We have recently had opportunities for testing the new truss, manufactured by Dr. Sheldon of New York. We have applied it in several cases in which other trusses have entirely failed to keep the hernia in place, but we have always succeeded with Dr. Sheldon's. It is the only truss we have ever seen that is made upon correct principles. It can be worn without discomfort and *invariably* answers the purpose for which it is intended. This is no *puff* for Dr. Sheldon's truss, but the plain facts in the case, and we take great pleasure in recommending it to the public. Dr. Sheldon's address is 710 Broadway, N. Y.

